# Tacrolimus-Induced Gallstone Formation in the Early Post-renal Transplant Period: A Case Report

**DOI:** 10.7759/cureus.85409

**Published:** 2025-06-05

**Authors:** Sutanay Bhattacharyya, Vipul Gupta

**Affiliations:** 1 Nephrology, Neotia Getwel Multispecialty Hospital, Siliguri, IND; 2 Nephrology, Fortis Hospital, Ludhiana, IND

**Keywords:** acute cholecystitis, gallstone disease, immunosuppression, renal transplant, tacrolimus

## Abstract

Gallstone disease (GSD) is a known but relatively underrecognized complication in renal transplant (RT) recipients, particularly in those receiving cyclosporine. However, there is scarce data on the association between tacrolimus use and cholelithiasis. We present the case of a 33-year-old female with end-stage renal disease who underwent an ABO-compatible RT and was initiated on a standard triple immunosuppressive regimen consisting of tacrolimus (3.5 mg/day), mycophenolate mofetil (1.5 g/day), and prednisolone (20 mg/day). Six weeks post-transplant, she developed acute abdominal pain, nausea, and right upper quadrant tenderness with a positive Murphy’s sign. Abdominal ultrasonography revealed multiple gallstones, the largest measuring 8 mm. She was managed conservatively and notably lacked traditional risk factors for gallstone formation. Pre-transplant imaging had not revealed any biliary abnormalities. Tacrolimus as a cause of GSD remains underreported. This report highlights the need for increased clinical awareness and further research to understand its potential role in post-transplant biliary complications.

## Introduction

Cholelithiasis is a relatively common complication among renal transplant (RT) recipients, with its prevalence being higher than in the general population. The usual risk factors for gallstone disease (GSD)- including gender, dyslipidemia, diabetes, and fatty liver - remain consistent in RT recipients. However, the higher prevalence in this population is linked to the use of immunosuppressive medications, the most common of which has been attributed to cyclosporine [1-3]. The role of tacrolimus in predisposing RT recipients to cholelithiasis, however, remains unclear. We present the case of an RT patient with normal ultrasonographic findings pre-transplant, who developed tacrolimus-related GSD as early as six weeks post-transplant.

## Case presentation

A 33-year-old female presented to our outpatient department and was diagnosed with end-stage kidney disease (ESKD) and advanced azotemia, with imaging showing bilaterally contracted kidneys. Due to the presence of uremic symptoms, she was initiated on maintenance hemodialysis via a right-sided tunneled catheter. She was subsequently enrolled in the RT program at our hospital. Baseline abdominal ultrasonography was normal, and a repeat scan performed one week before transplantation also showed no abnormalities. After routine pre-transplant evaluation, she underwent an ABO-compatible, HLA 10/12-matched live donor RT, with her mother as the donor. No induction therapy was given. Post-transplant, she demonstrated immediate graft function with a urine output of approximately 7 liters per day. She was discharged in a stable condition with a serum creatinine of 1.2 mg/dl. Her immunosuppressive regimen consisted of tacrolimus (3.5 mg/day), mycophenolate mofetil (1.5 g/day), and prednisolone (20 mg/day).

**Figure 1:**
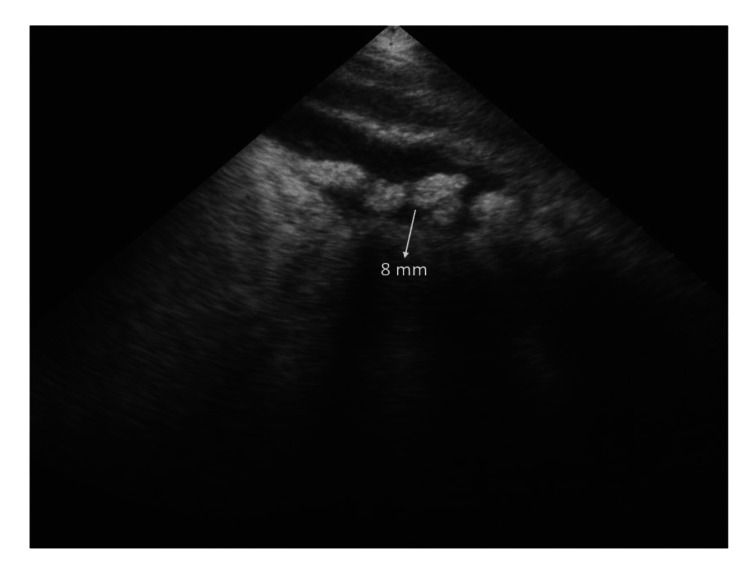
Ultrasound image showing multiple gallstones in the gallbladder

Approximately six weeks post-transplant, the patient presented with a two-day history of abdominal pain, dehydration, and marked tenderness with guarding in the right hypochondrium, along with a positive Murphy’s sign. These clinical features were suggestive of early acute cholecystitis. She was started on intravenous fluids, analgesics, and empirical antibiotics: ceftriaxone 1 g twice daily and metronidazole 500 mg intravenously three times daily. Abdominal ultrasonography revealed multiple gallstones, with the largest measuring 8 mm, without evidence of gallbladder wall thickening or pericholecystic fluid. A surgical consultation confirmed the diagnosis of acute cholecystitis. She responded well to conservative management and was discharged in a stable condition with a plan for elective cholecystectomy (Figure 1).

Her tacrolimus trough level was elevated at 13.6 ng/ml, which was confirmed on repeat testing from the same lab. Her dose was subsequently reduced from 3.5 mg/day to 3 mg/day. A repeat trough level after three days was 8.8 ng/ml. Clinically, she was non-obese with a BMI within the normal range. Her laboratory findings, summarized in Table 1, revealed normal lipid and liver enzyme levels, with no evidence of diabetes or pancreatitis.

**Table 1:** Summary of laboratory investigations at the time of presentation ALP: alkaline phosphatase; ALT: alanine transaminase; AST: aspartate aminotransferase; BMI: body mass index; HDL: high-density lipoprotein; LDL: low-density lipoprotein; SGOT: Serum glutamic-oxaloacetic transaminase; SGPT: serum glutamic-pyruvic transaminase

Parameter	Result	Reference range	Units
Serum creatinine	1.2	0.6 – 1.3	mg/dL
Tacrolimus trough level	13.6	5 – 12	ng/mL
BMI	22.3	18.5 – 24.9	kg/m²
Total cholesterol	156	<200	mg/dL
LDL cholesterol	82	<130	mg/dL
HDL cholesterol	48	>40	mg/dL
Triglycerides	112	<150	mg/dL
Blood glucose (fasting)	92	70 – 100	mg/dL
AST (SGOT)	22	10 – 40	U/L
ALT (SGPT)	26	7 – 56	U/L
ALP	86	44 – 147	U/L
Serum amylase	54	30 – 110	U/L
Serum lipase	32	23 – 300	U/L

Tacrolimus levels were elevated during presentation and normalized after dose adjustment. The patient was subsequently discharged and followed up in the outpatient transplant clinic, where she remained asymptomatic and was advised to undergo elective cholecystectomy.

## Discussion

GSD is an uncommon but clinically relevant complication in RT recipients. While the association of cyclosporine-based immunosuppressive therapy has been well documented, reports of tacrolimus-related cholelithiasis are exceedingly rare, especially in the early post-transplant period. Our patient developed symptomatic GSD just six weeks after RT, raising the possibility of a causal relationship with tacrolimus. The exact prevalence of gallstones in RT recipients is not well established, but studies have reported rates ranging from 17% to 20% [4,5]. The use of cyclosporine as a part of the immunosuppressive regimen has been recognized as a contributory factor to the development of cholelithiasis. Although the exact mechanism by which cyclosporine causes gallstone formation is not fully understood, studies in both humans and animals suggest that its metabolism in the liver and predominant excretion through bile may impair bile salt secretion, leading to cholestasis, a key factor in stone formation [6]. Tacrolimus, like cyclosporine, is a calcineurin inhibitor (CNI) and is hypothesized to increase the risk of gallstone formation in transplantation recipients. Animal model studies showed that although tacrolimus increased bile secretion, it has little influence on bile flow, a factor that may contribute to stone formation [7,8].

In our patient, pre-transplant evaluation did not reveal any of the well-known risk factors for the development of gallstones. Routine abdominal ultrasonography on multiple occasions, including a scan performed just one week before RT, showed no evidence of cholelithiasis. However, within two weeks post-transplant, she developed full-blown symptoms of acute cholecystitis secondary to gallbladder calculi. During this short course of time, she did not develop any of the traditional risk factors for cholelithiasis, including dyslipidemia and new-onset diabetes. Therefore, the only causal etiology left was drug-related. An extensive literature search did not reveal any pathophysiological mechanisms linking gallstone formation to mycophenolate mofetil and steroids. None of the other non-immunosuppressive medications, including cotrimoxazole and ranitidine, have been implicated in affecting gall bladder function to date. Notably, the patient had elevated trough tacrolimus levels during the episode, which led us to consider tacrolimus as an etiology of GSD in this patient.

A notable aspect of this case is the unusually early development of gallstones following transplantation. Our patient developed symptomatic cholelithiasis within just six weeks post-transplant. In contrast, Alberú et al. reported a higher prevalence of gallstones in kidney transplant recipients who had been on cyclosporine for more than 24 months, suggesting that gallstone formation typically occurs later in the post-transplant course [3]. Similarly, Lorber et al. reported the earliest onset of gallstones at eight months post-transplant in patients receiving cyclosporine-based immunosuppression [9]. This clear difference raises the possibility that tacrolimus may have a distinct or accelerated effect on biliary physiology. It is also possible that biliary sludge developed initially and rapidly progressed to stone formation, as suggested by mechanisms described in animal models. The development of acute cholecystitis in this patient further emphasizes the potential for early and clinically significant gallstone-related complications in this subset of transplant recipients.

To the best of our knowledge, this is the first reported case of tacrolimus-related cholelithiasis in an RT recipient, not just among the Indian population, but also worldwide. Also, the very early development of this condition is another finding that needs to be taken into account. However, a definitive causal relationship cannot be established based on a single case. Large population-based studies need to be conducted to explore this association further.

## Conclusions

Tacrolimus as an etiology or predisposing factor for early-onset cholelithiasis needs to be kept in mind when evaluating RT recipients. Routine ultrasonographic screening might be an effective option to identify at-risk individuals, as these patients may be more susceptible to cholelithiasis-related complications. In selected cases, elective cholecystectomy could be considered as a preventive strategy.
